# Role of Ajwa Date Fruit Pulp and Seed in the Management of Diseases through In Vitro and In Silico Analysis

**DOI:** 10.3390/biology11010078

**Published:** 2022-01-05

**Authors:** Shehwaz Anwar, Ravindra Raut, Mohammed A. Alsahli, Ahmad Almatroudi, Hani Alfheeaid, Faisal M. Alzahrani, Amjad Ali Khan, Khaled S. Allemailem, Saleh A. Almatroodi, Arshad Husain Rahmani

**Affiliations:** 1 Department of Medical Laboratories, College of Applied Medical Sciences, Qassim University, Buraydah 51452, Saudi Arabia; shehwazanwar25@gmail.com (S.A.); shly@qu.edu.sa (M.A.A.); aamtrody@qu.edu.sa (A.A.); smtrody@qu.edu.sa (S.A.A.); 2 Department of Biotechnology, National Institute of Technology Durgapur, Durgapur 713209, India; raviraut206@gmail.com; 3 Department of Food Science and Human Nutrition, College of Agriculture and Veterinary Medicine, Qassim University, Buraydah 51452, Saudi Arabia; h.alfheeaid@qu.edu.sa; 4 Department of Clinical Laboratory Sciences, College of Applied Medical Sciences, Imam Abdulrahman Bin Faisal University, Dammam 31441, Saudi Arabia; fmzahrani@iau.edu.sa; 5 Department of Basic Health Sciences, College of Applied Medical Sciences, Qassim University, Buraydah 51452, Saudi Arabia; akhan@qu.edu.sa

**Keywords:** Ajwa, antidiabetic, anti-inflammatory, antioxidant, anti-hemolytic, oxidative stress, molecular docking

## Abstract

**Simple Summary:**

Most diseases result in an imbalance of antioxidant defense, inflammatory responses, and membrane permeabilization. The current therapeutic modules of disease prevention are not fully effective and have some adverse effects on physiological parameters. In this vista, medicinal plants and their active compounds have proven to be effective against disease prevention and treatment. Ajwa dates have high nutritional value and are reported to possess antioxidant, anti-inflammatory, and antitumor properties. In the current in vitro study, Ajwa fruit pulp and seed extract were found to have strong antioxidant properties, stabilize the RBC membrane, and have a good protective capacity against protein denaturation. Besides this, the seed extract prevents glucose-mediated browning of BSA as well as inhibiting the development of cross-amyloid and AGEs formations. Molecular docking results confirm the interaction between functional residues of antioxidant enzymes and components of Ajwa fruit pulp and seed contents. Therefore, the consumption of Ajwa dates can be beneficial in disease prevention and treatment. However, more detailed study is required based on pharmacological aspects to determine the mechanisms of action of Ajwa dates’ components in disease prevention.

**Abstract:**

This study investigated the health-promoting activities of methanolic extracts of Ajwa date seed and fruit pulp extracts through in vitro studies. These studies confirmed potential antioxidant, anti-hemolytic, anti-proteolytic, and anti-bacterial activities associated with Ajwa dates. The EC_50_ values of fruit pulp and seed extracts in methanol were reported to be 1580.35 ± 0.37 and 1272.68 ± 0.27 µg/mL, respectively, in the DPPH test. The maximum percentage of hydrogen peroxide-reducing activity was 71.3 and 65.38% for both extracts at 600 µg/mL. Fruit pulp and seed extracts inhibited heat-induced BSA denaturation by 68.11 and 60.308%, heat-induced hemolysis by 63.84% and 58.10%, and hypersalinity-induced hemolysis by 61.71% and 57.27%, and showed the maximum anti-proteinase potential of 56.8 and 51.31% at 600 μg/mL, respectively. Seed and fruit pulp inhibited heat-induced egg albumin denaturation at the same concentration by 44.31 and 50.84%, respectively. Ajwa seed showed minimum browning intensity by 63.2%, percent aggregation index by 64.2%, and amyloid structure by 63.8% at 600 μg/mL. At 100 mg/mL, Ajwa seed extract exhibited good antibacterial activity. Molecular docking analysis showed that ten active constituents of Ajwa seeds bind with the critical antioxidant enzymes, catalase (1DGH) and superoxide dismutase (5YTU). The functional residues involved in such interactions include Arg72, Ala357, and Leu144 in 1DGH, and Gly37, Pro13, and Asp11 in 5YTU. Hence, Ajwa dates can be used to develop a suitable alternative therapy in various diseases, including diabetes and possibly COVID-19-associated complications.

## 1. Introduction

Inflammation and hyperglycemia have been found to be two significant pathologies associated with the severity of various diseases and increasing death rates across the world. All the diseases are linked with increased inflammatory biomarkers and cytokines [1]. Insulin resistance, hyperglycemia, and cell death are all caused by changes in the shape and function of β cells and endothelial cells as a consequence of cytokine release [2]. In addition, diseases involving severe liver tissue damage decrease glycogen production and increase insulin tolerance and hyperglycemia. As a result, treating hyperglycemia will result in a decrease in serum levels of cytokine [3].

The accumulation of different reactive oxygen species (ROS) is an indicator of oxidative stress [4]. ROS are very active oxidant molecules with an additional electron. Overproduction of superoxide ions leads to activation of five key pathways linked to diabetic complications, such as polyol pathway flux, protein kinase C activation, agitation of the hexosamine pathway, increased production of advanced glycation end products (AGEs), as well as increased expression of AGE receptors and their activating ligands [5]. Constant hyperglycemia along with diabetic conditions damages various organs and leads to various macrovascular complications such as premature atherosclerosis, myocardial infarctions, peripheral vascular disease, as well as microvascular complications such as nephropathy, neuropathy, and retinopathy. AGEs are covalent products of nonenzymatic glycation and oxidation of various biomolecules [6]. AGEs have been reported to be involved in the pathology of various diseases such as diabetes, inflammation, neurodegeneration, and aging. Thus, checking the formation and accumulation of AGEs can be a plausible move for treatment/prevention of these disorders [7]. 

A complex system of antioxidant molecules and enzymes works provides protection against oxidative stress by preventing the excess of ROS, neutralizing the free radicals, and restoring the damage caused by ROS. It has been established that damage to this defense system, and, hence, increased oxidative stress, has been linked to various disorders [8] and pathogenesis, mortality risk, and severity in patients with SARS-CoV infection [9]. Glycation-mediated inactivation of antioxidant enzymes such as superoxide dismutase can disrupt the cellular antioxidant defense system that contributes to a variety of pathologies linked with long-term diabetic complications [10,11,12,13,14].

While numerous off-label medicines have shown encouraging results in the treatment of so many commonly found diseases, there is currently no complete licensed therapy possible to treat these diseases and disorders. As a result, there is a great need for possible medical remedies or the use of off-label medicines in this situation. As a result, it is important to investigate alternative medicine, such as folk medicine, as an alternative to traditional diabetic treatments and other related diseases. Herbal drugs with antioxidant potential and scientifically proved benefits in the treatment of diabetes mellitus may be recommended as a complementary therapy of traditional treatments for COVID patients [15]. 

**Figure 1:**
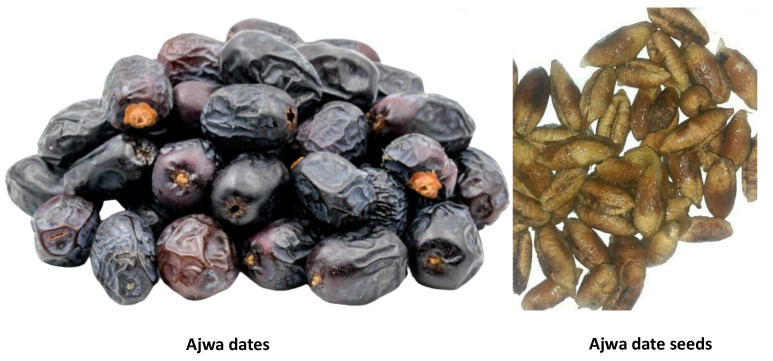
Ajwa dates and their seeds.

Different varieties of date palm (*Phoenix dactylifera*) are consumed in Arab areas for centuries due to their excellent nutritional properties. Ajwa dates from Saudi Arabia have been documented to be a rich source of antioxidants and other nutritious components [16] (Figure 1). Besides, date palms have been well claimed to possess potent hepato-protective, nephroprotective, anticancer, antidiabetic, anti-ulcerative, and antihypertensive properties. Moreover, all varieties of date including Ajwa have exhibited antioxidant and antitumor properties [17]. 

Molecular docking is indeed a type of in silico modeling in which two or more molecules combine to form a stable adduct. Docking suggests the three-dimensional structure of any complex based on the binding characteristics of target molecules (protein, DNA, etc.) and ligand. It generates a variety of potential adduct structures, which are scored and classified using the software’s scoring algorithm. Based on the overall energy of the system, docking simulations indicate the best suitable docked conformers. The goal is to predict the bound conformations and the binding affinity [18].

The goal of our study was to investigate in vitro health beneficial capabilities of methanolic extract of Ajwa date seed and fruit pulp to explore a possible alternative strategy against the inhibition of pathogenesis mortality of diabetic patients afflicted with COVID-19. The molecular docking studies with superoxide dismutase and catalase and Ajwa compounds were conducted to investigate possible antioxidant potential of Ajwa in preventing oxidative stress and relate the most promising molecules of Ajwa to their chemical characteristics.

## 2. Materials and Methods

### 2.1. Materials

Trichloroacetic acid, trypsin, 2,2-diphenyl-1-picrylhydrazyl (DPPH), gallic acid, ferric chloride, Folin–Ciocalteu reagent, ascorbic acid, potassium ferricyanide, quercetin, and Congo red were obtained from Sigma Co. St. Louis, MO, USA. Disodium hydrogen phosphate, hydrochloric acid, DMSO, mono sodium dihydrogen phosphate, aluminum chloride, ethanol, methanol, sodium carbonate, sodium hydroxide, and hydrogen peroxide have been received from Merck, Darmstadt, Germany. Analytical-grade chemicals and reagents were used, whereas the solvents were of HPLC grade in current study. 

### 2.2. Preparation of Extracts

The methanol extracts of Ajwa seed and fruit pulp were obtained following our standardized procedure. In a nutshell, the seeds and pulp were detached and properly cleaned with distilled water. They were dried thoroughly afterwards. To create the powder, the dry ingredients were finely ground. To grind the seed, a mortar and pestle were employed followed by use of an electronic mixer grinder. The methanol samples were obtained via soaking approximately 100 g of plant stuff in powder form independently in 1 liter of 97% methanol, in a magnetic shaker over three hours at 37 °C. The extracts were purified by filtration before being condensed using rotary evaporators at decreased pressure and 40 °C to obtain raw active component. Both extracts were kept at 4 °C for future analysis. The % yield of extract was calculated using the equation below [19].

Percent yield= [Weight of sample extract/Starting weight of sample] × 100

### 2.3. Phytochemical Screening

A previously published paper was used to conduct phytochemical screening of carbohydrates, phenolics, alkaloids, saponins, flavonoids, tannins, anthraquinone, as well as phenolic compounds [20]. Carbohydrate detection was accomplished through mixing 1000 µL of iodine solution and 3000 µL of seed and fruit pulp extracts prepared in methanol separately. Carbohydrates could be confirmed if the color turned to purple. The existence of saponins was determined by mixing 5 mL of distil water with 5 mL of extracts in a test tube and aggressively shaking the mixture before warming the test tube. The presence of saponins is indicated by the development of stable froth. Meanwhile, 2000 µL of extracts and 2000 µL of distil water were mixed in a vial. The condensed tannins were indicated by the formation of green precipitate after adding a few drops of FeCl_3_. The availability of flavonoids in both extracts were verified by that of the development of a strong yellow color when 2 mL of extracts were mixed with a 20% sodium hydroxide solution. However, after the addition of dilute HCl, the yellowish color becomes colorless. When a few drops of FeCl_3_ were combined with the extracts, the development of such a blue black color reported the existence of phenolic chemicals.

### 2.4. Evaluation of Phenolic Content

Within this experiment, extracts of seed and fruit pulp (0.500 mL, 1000 µg/mL) were placed separately in various test tubes containing 2500 µL of Folin-Ciocalteu reagent (10%). Then, 2000 µL of sodium carbonate (7.5%) was eventually added to all tubes. The tubes were maintained at 37 °C in the absence of light for 30 min. After incubation, the absorbance of various solutions was recorded with a spectrophotometer at 760 nm. Various concentrations of gallic acid (50–250 g/mL) were used to construct a standard curve plot. The phenolic contents of both extracts were calculated using this standard curve. Phenolic contents of extracts were evaluated in milligram (GAE). All of the experiments tests were conducted twice. The results were expressed as mg equivalents of gallic acid per gm sample extract. The following formula was used to calculate total phenolic content [19]:

Total phenolic content= K × Vol/w

where Vol is the volume (mL) of sample used in the extraction. K is the concentration of gallic acid in mg/mL, and w is the weight of pure dried sample used (g). 

### 2.5. Evaluation of Flavonoid Contents in Extracts

The flavonoid contents of both extracts were estimated using the method described in our prior article [19]. Quercetin (20, 25, 50, 75, 100, 150, 200, and 250 µg/mL) was employed to create a standard plot. In a nutshell, 500 µL of quercetin solution or extract (50 µg/mL) were added to a vial having AlCl_3_ solution (500 µL, 2%). Intermittent mixing of the vials was done. The absorbance of each solution was measured at 420 nm spectrophotometrically against methanol (blank) after 1 h of incubation of tubes at room temperature. Flavonoid contents of extracts were expressed as milligram quercetin equivalent per gram sample extract (mg QUE/g). To evaluate the flavonoid contents of both extracts, the following equation was employed:

TFC = K × Vol/w

where Vol is the volume of plant extract (mL). K is the concentration of quercetin (mg/mL), and w is the weight of the pure dried sample used (g).

### 2.6. Reducing Capacity Assessment

This assay was performed as per the method described in prior articles with slight changes [19,20]. Actually, it measured the potential of extracts to convert more oxidized ferric ions into lesser oxidized ferrous ions at room temperature. This reaction resulted in the change of absorbance monitored at 700 nm for both control and test samples against phosphate buffer (pH 6.6) only. All of the assays were done thrice. The percentage reducing capacity was calculated using the formula provided below [19].

Percentage reducing ability= [(Zc − Zs)/Zc] × 100

where Zc denotes the absorbance of the control (solution not having any extract) and Zs indicates the absorbance of extract containing solution. 

### 2.7. Scavenging of Hydrogen Peroxide (H_2_O_2_)

This activity was investigated as per the method described in prior researchers with slight changes [19,21]. The extracts (100, 200, 300, 400, 500, and 600 μg/mL) or ascorbic acid were mixed separately with 1000 µL of H_2_O_2_ solution (40 mM prepared in phosphate buffer pH 7.4). The absorbance of all samples was determined at 230 nm after 10 min of incubation of tubes against phosphate buffer. All the experiments were done three times. The hydrogen peroxide percentage scavenging potential was then evaluated using the following equation. The percentage of scavenged H_2_O_2_ was calculated by the formula provided below [19].

H_2_O_2_ scavenging ability (%) = [(Zc − Zs)/Zc] × 100

where Zc indicates the absorbance of H_2_O_2_ solution. Zs denotes the absorbance of the solution having both H_2_O_2_ and extract.

### 2.8. DPPH Assay

The antioxidant activity was further confirmed on the basis of scavenging 1,1 difenyl-2-picryl-hydrazyl (DPPH) as described in prior articles [19,22,23]. Briefly, 2.5 mL of increasing concentration of methanolic extracts (50, 100, 200, 300, 400, 500, and 600 μg/mL) was mixed with DPPH (1 mL, 0.3 mM; prepared in analytical grade methanol). The absorbance of each solution was noted at 517 nm followed by 30 min incubation of each solution against methanol. However, control contained DPPH in methanol for this assay.

Percentage DPPH scavenging activity = [(Zc − Zs)/Zc] × 100)

where Zc indicates the absorbance of solution without extract. Zs denotes the absorbance of the solution having extract. 

### 2.9. Albumin Denaturation Inhibition Activity

Evaluation of anti-inflammatory potential of both extracts was evaluated by inhibition of albumin denaturation as described earlier [19,24,25] with slight modifications. Ibuprofen was used as a reference drug for this experiment. The test solution contained 500 μL of 1% aqueous solution of bovine serum albumin (BSA) as well as 100 μL of extracts (100, 200, 300, 40, 500, and 600 µg/mL) or 200 µg/mL of non-steroidal anti-inflammatory drug ibuprofen in separate tubes. Samples were taken out from the incubator after incubation for 20 min at 37 °C. The solutions were heated at 71 °C for 10 min to denature the protein. After cooling, the turbidity of samples was spectrophotometrically recorded at 660 nm, against distil water. The tests were done three times. The inhibition of BSA denaturation (%) was determined using the formula provided below [19].

Percent inhibition= [(Zc − Zs)/Zc] × 100

where Zc indicates the absorbance of control. Zs indicates the absorbance of the sample containing extract or ibuprofen.

### 2.10. Inhibition of Proteinase Action

The methodology followed by Anwar et al. (2020a) [19] and Sakat et al. (2010) [25] was used to test the inhibitory action of Ajwa date on trypsin. The testing solutions (2000 µL) had 1000 µL of tris HCl buffer (20 mM; pH 7.4), trypsin (60 µg), and 1000 µL of varying concentration of extracts (100, 200, 300, 400, 500, and 600 μg/mL) or 0.20 mg/mL of diclofenac sodium. After 5 min at room temperature, 1000 µL of casein (0.8%) was added to the tubes. The tubes were kept at room temperature for 5 min, and 1 mL of casein (*w*/*v*) was added. To cease the reaction, 2000 µL of 70% perchloric acid was included after 20 min that resulting in the formation of a cloudy solution. The centrifugation was carried out at 2500 rpm for 5 min. At 210 nm, the supernatant’s absorbance was tested against a blank containing just a buffer. Triplicates of each sample were taken.

Percentage inhibition of proteinase action (%) = [(Zc − Zs)/Zc] × 100

where Zc signifies the absorbance of a control sample, and Zs denotes the absorbance of the sample having either the extract or diclofenac.

### 2.11. Inhibition of Egg Albumin Denaturation

Phosphate buffer saline (2800 µL, pH 6.4), raw egg albumin of hen (200 µL), and 2000 µL of different concentrations (100, 200, 300, 400, 500, and 600 µg/mL) of extracts were taken in the reaction solution (5 mL) [24,25]. Diclofenac sodium was used as a standard drug (200 µg/mL). In a BOD incubator, various tubes containing these solutions were kept for 15 min at 37 ± 2 °C. The reaction mixtures were then heated for 5 min at 70 °C. After cooling, their absorbance was taken at 660 nm spectrophotometrically against buffer phosphate saline. The formula provided below was used to determine the percentage egg albumin denaturation inhibition.

Percentage Inhibition = [(Zc − Zs)/Zc] × 100(1)

where Zc signifies the absorbance of a control sample, and Zs denotes the absorbance of the sample having either the extract or diclofenac.

### 2.12. Assessment of Potential of Membrane Stabilization

*a*  *Preparation of Red Blood Cell (RBC) Suspension*

A healthy and fit volunteer gave a fresh blood sample after fourteen days of not using any anti-inflammatory (non-steroidal) or anti-contraceptive drugs. The blood samples were then shifted to vials containing the same amount of sterilized Alsever’s solution as the blood sample. The resulting solutions were subsequently centrifuged for 10 min at 3000 rpm. The erythrocyte sediments were washed three times using an equal volume of normal saline after the discarding of supernatant plasma. The volume of each resultant was determined and isotonic phosphate buffer (pH 7.4) was employed to reconstitute RBC suspension (10% *v*/*v*) [19,26].

*b*  *Heat Induced Hemolysis*

Heat-induced erythrocyte hemolysis was carried out as detailed in our earlier articles [19] with a few changes. Then, 1000 µL of extract (100, 200, 300, 400, 500, and 600 µg/mL) or aspirin (200 µg/mL) was combined with 1000 µL of previously prepared RBC solution (10% *v/v*). For 20 min, all of the tubes were kept in a water bath at 56 °C. After incubation, different tubes were removed from the water bath and were cooled. The tubes were centrifuged for 5 min at 2500 rpm and 4 °C. At 540 nm, the absorbance of resultant supernatants was measured using a spectrophotometer. The control had buffer and RBC suspension (without drug or extract), whereas the blank used phosphate buffer only. The percentage protection against hemolysis induced by heat was estimated using the formula provided below.

Percentage protection = [(Zc − Zs)/Zc] × 100(2)

where Zc signifies the absorbance of a control sample, and Zs denotes the absorbance of the sample having either the extract or aspirin.

*c*  *Inhibition of Hyposaline Induced Hemolysis*

Hyposaline-induced erythrocyte hemolysis was carried out as detailed by earlier articles [19,26] with a few changes. Then, 1000 µL of phosphate buffer (pH 7.4, 0.1 M), hyposaline (2000 µL), and 500 µL RBC suspension (10% *v*/*v*) were mixed with 1 mL of varying concentration of extracts (100, 200, 300, 400, 500, and 600 μg/mL) or diclofenac (200 µg/mL). The control solution, on the other hand, used distil water in the place of hyposaline, and there was neither drug nor extract. At 37 °C, all tubes were incubated for 30 min. After the incubation period, the tubes were centrifuged for 10 min at 3000 rpm. At 560 nm, the absorbance of the collected supernatant was determined. By assuming 100% hemolysis in the control, the percent protection from hyposalinity-induced hemolysis was evaluated [19].

Percentage protection = 100 − [(Zs/Zc) × 100](3)

where Zc signifies the absorbance of a control sample, and Zs denotes the absorbance of the sample having either the extract or diclofenac. 

### 2.13. Screening of Antiglycating and AGEs Formation Inhibiting Potential

*a*  *Incubation of Extracts with In Vitro Glycation System*

Brownlee’s methodology was applied with minor changes [27]. Only Ajwa seed extract was used in antiglycation and AGEs formation inhibition study. In this experiment, glucose (500 mM) and BSA (10 mg/mL) were combined with or without Ajwa seed extract (100, 200, 300, 400, 500, and 600 μg/mL) in phosphate buffer (0.1 M, pH 7.4). The mixtures were stored at room temperature for 15 days on a shaker away from direct sunlight. The incubated samples were subsequently dialyzed overnight at 37 °C against phosphate buffer (50 mM, pH 7.4) to eliminate unbound glucose. The dialysis of the incubated samples was done to eliminate unbound glucose at 37 °C using phosphate buffer (50 mM, pH 7.4) for the whole of the night. The concentration of BSA in each sample was calculated using the molar extinction coefficient. The samples were immediately kept at −20 °C for further use. Each sample was treated with 3 mM/L of sodium azide to avoid bacterial contamination. All of the trials were repeated three times.

*b*  *Assessment of Browning Intensity*

Glycation has been reported to have a significant role in diabetes and its complications [4]. The browning intensity of glycated samples has been found to be an indicator of glycation of various samples [19]. The intensity of browning was screened by recording the absorbance of different glycated samples at 420 nm [28] using a 1-cm path length cell after diluting with distill water. All the experiments were carried in triplicates. The relative percentage browning intensity was determined by the formula given below [19].

Percentage protection from browning = [(Zc − Zs)/Zc] × 100

where Zc indicates the absorbance of glycated samples without extract. Zs denoted absorbance of glycated samples having seed extract.

*c*  *Effect on Protein Aggregation Index*

Glycation induces the structural alterations in biomolecules such as proteins. The structural abnormality may lead to the formation of aggregates. Protein aggregate formation is further linked to various diseases and their complications. Therefore, it is very necessary to investigate the protective activity of natural products against glycation induced protein aggregates formation to develop a strategy for the treatment and management of diseases linked with aggregate formation. The protective ability of Ajwa seed extract was investigated by recording the absorbance of different glycated samples either having seed extract (100, 200, 300, 400, 500, and 600 µg/mL) or not having seed extract. The aggregation index was calculated by the following formula

Percentage of protein aggregation index = [A_340_/(A_280_ − A_340_)] × 100

where A_340_ = Absorbance at 340 nm and A_280_ = Absorbance at 280 nm.

*d*  *Percent Inhibition of Fibrillar State: Congo Red Assay*

Congo red assay was performed to evaluate the percent inhibition of glycation-induced fibrillation of BSA. The CR assay is based on the capacity of dye binding with fibrils [29]. Congo red (amyloid specific dye) was prepared according to previously published articles [19,28]. The absorbance was measured for different samples such as AGE-BSA with seed extract only (100, 200, 300, 400, 500, and 600 μg/mL), AGE-BSA, and native BSA separately, as well as for the background of Congo red. In short, 500 µL of glycated protein solution/native BSA (100 μM), and 100 μL of Congo red (100 μM) were incubated at room temperature for 10 min. The absorbance of each sample was measured at 530 nm.

% inhibition of amyloid formation= [(Zc − Zs)/Zc] × 100

where Zc is the measured absorbance of BSA and glucose system that does not have extract. Zs is the absorbance of BSA and glucose system incubated with seed extract or BSA not incubated with glucose or extract.

### 2.14. Antimicrobial Activity

The antimicrobial potential of extracts was evaluated using five strains of bacteria and one strain of fungal. Gram-positive cocci included *Enterococcus faecalis* ATCC 29212 and *Staphylococcus aureus* ATCC 29213. Gram-negative bacilli listed *Escherichia coli* ATCC 25922, *Pseudomonas aeruginosa* ATCC 27853, and *Klebsiella pneumonia* ATCC 700603). All strains were obtained as a gift from the Microbiology lab, College of Applied Medical Sciences, Qassim University, and KSA.

### 2.15. Dilutions and Inoculum Preparations

Bacterial and fungal inoculum from fresh pure cultures were prepared using Muller Hinton broth, and 0.5 McFarland standard was used for comparison of each bacterial and fungal suspension. Two concentrations of extracts (25 and 50 mg/mL) were prepared by serial dilution of seed and pulp extracts stock solution in sterile distilled water.

### 2.16. Procedure for Performing the Well Diffusion Test

Agar well diffusion assay was performed to investigate the antimicrobial activity against the selected microorganisms [20]. A sterile cotton swab was used for the spreading procedure. The backside of blue micropipette tips was used to make two wells in the agar plate with the bottom of wells having melted MHA agar for sealing. Two different concentrations of extracts were included in different wells. The positive controls were Erythromycin 15, Vancomycin 30, Imipenem 30, Amikacin 30, and Fluconazole 10. After incubation for 1 day at 37 °C, the zone of inhibition around the wells was investigated. A ruler was used to record the zone diameter in millimeters (mm).

The lowest antimicrobial concentration that completely inhibited the bacterial growth in all replicate wells was used to calculate the MIC (minimum inhibitory concentration). A plot of bacterial growth vs. extract concentration was found very steep to estimate IC50 accurately. As a result, the MIC was employed for bacteria in this experiment. By dissolving the extract in nutritional broth, several quantities of Ajwa seed extracts (500, 250, 125, 100, 50, 25, and 6.25 mg/mL) were prepared individually. The bacterial solution was adjusted to 1.5·10^−8^ CFU at McFarland standard and diluted to 1:100. To calculate the MIC, 50 µL of the various extract concentrations was mixed with 50 µL of the bacterial suspensions on sterile 96-well plate nutrient agar (Oxoid) (Corning Costar Ltd., Tewksbury, MA, USA). The plates were then incubated for 16 to 20 h at 35 °C. The MIC was determined by taking the lowest concentration of extract in the well with no bacterial growth (indicated as no turbidity).

### 2.17. Statistical Analysis

All the experiments were carried out in triplicate, expressed as means ± standard error. The statistical analysis was performed using ANOVA. The probability, *p* < 0.05 was considered as statistically significant for a test.

## 3. Docking Studies

### 3.1. The Receptors

For the present study, two very important enzymes of the cellular anti-oxidant mechanism, viz. catalase, and superoxide dismutase (SOD), were selected for the prediction of possible interaction of the constituents and metabolites of Ajwa date seed extract. Any interaction between residues of enzymes and these constituents might contribute to possible protection against glycation or oxidative stress-induced denaturation of these enzymes. Three-dimensional structures of human erythrocyte catalase 3-amino-1,2,4-triazole complex (PDB id: 1DGH) human SOD 1 complexed with isoproterenol (PDB id: 5YTU) were downloaded from the Protein Databank website (https://www.rcsb.org/, last accessed on 18 June 2021) in the .pdb formats. The two structures are crystal structures determined using X-ray diffraction at resolutions of 2 and 1.9 Å, respectively. Selection of the structures was based on resolution, source organism, and availability of bound ligand for reference of the active site, and the residues of the active site.

### 3.2. The Ligands

It was found that 3,30-di-O-methyl ellagic acid, 7-methoxyquercetin-O-hexose isomers, caffeic acid, ferulic acid, quercetin-rutinoside, 6′′′-malonylicariin, p-hydroxybenzoic acid, phytol, punicalagin, and quercetin-3-O-glucoside (isoquercitrin) are active constituents of date fruit. Three-dimensional structures of all the ligands were downloaded from the NCBI PubChem compound database (https://pubchem.ncbi.nlm.nih.gov/, last accessed on 18 June 2021) in the .sdf formats. Details of the ligands with their properties are provided in the result section with the sub-heading Receptor–Ligand Interaction Study by Molecular Docking.

Since three-dimensional conformers of 6′′′-malonylicariin and punicalagin are not available in the NCBI PubChem compound database, two-dimensional conformers, bearing ID 135398032 and 44584733, were downloaded from the database. Then these structures were converted to three-dimensional conformers using Web-based SMILES Translation Service; Online SMILES Translator and Structure File Generator (https://cactus.nci.nih.gov/translate/, last accessed on 18 June 2021). 

### 3.3. Molecular Docking

Molecular docking is a powerful computational modeling tool in evaluating the binding of a ligand (phytochemicals or others) to the active site of an enzyme or receptor. Molecular docking was performed for identifying the interaction between substrate and enzyme. The structure of ligands was collected from the PubChem database [30]. AutoDock vina [31] was used for docking, and the protein was prepared accordingly in BIOVIA Discovery Studio Visualizer (https://discover.3ds.com/discovery-studio-visualizer-download/, accessed on 18 June 2021) [32]. All water molecules available with the receptors were removed from the workspace.

The grid sizes of the docking were center_x = 25.591047, center_y = 40.102372, center_z = 60.221023 along the size 20 for X, Y, and Z-axis for 1DGH and center_x = −76.243300, center_y = 6.146800, center_z = −3.292600, respectively, along the size 20 for X, Y, and Z-axis for 5YTU, respectively. Before performing the docking experiment, polar hydrogens were added to each protein receptor, and Kollman’s partial atomic charges were applied to minimize the energy. The processed structure of receptors was saved in PDBQT file format, which contains hydrogens in all polar residues. MGL tools (https://ccsb.scripps.edu/mgltools/downloads/, last accessed on 20 June 2021) were used to process the receptor and ligands by adding hydrogen atoms [33]. The maximum number of runs was set to 8. The best docking in terms of free energy of binding (expressed as negative values) was considered for further analysis.

## 4. Results

Ajwa dates are consumed quite often in Gulf countries as they possess some excellent nutritious components with antioxidant properties. It has been reported that Ajwa dates play a significant role in the management of different diseases through the modulation of various biological activities. In this study, the role of Ajwa dates, pulp, and seeds in disease management was explored through in vitro and in silico analysis.

The DPPH approach is the most often used in vitro antioxidant activity measurement, whereas lipid peroxidation approach is the most widely utilized in vivo antioxidant activity evaluation [34]. The screening for antioxidant potential necessitates the use of methods that focus on the kinetics of reactions that include antioxidants and address the mechanism of antioxidant activity. It has been seen that methods based on suppressed autoxidation are best for termination-enhancing and chain-breaking antioxidants. On the other hand, distinct particular investigations are needed for preventative antioxidants [35]. Oxidative stress, systemic hyper-inflammatory reactions, glycation of biomolecules, advanced glycation end-product formation, and permeabilization of the lysosomal membrane are all very commonly linked to a range of diseases such as diabetes and its complications. Our study indicated the strong antioxidant potential of Ajwa dates that may link the therapeutic potential of the Ajwa date against oxidative stress, denaturation of proteins, stability of membranes, as well as AGE formation. Preliminary phytochemical analysis of the methanolic extract of Ajwa revealed the presence of different biomolecules.

### 4.1. Preliminary Screening, Flavonoid, and Phenolic Content

Ajwa dates are usually black in color, and are most commonly cultivated in Medina, Saudi Arabia (Figure 1). The color, odor, texture, and the yield (percentage) of methanol extracts of Ajwa seed as well as fruit pulp extract are provided in Table 1. The results of phytochemical screening of Ajwa fruit pulp and seed extract for the presence of different phytoconstiuents are provided in Table 2. Further, TPC in fruit pulp and seeds extracts were 245.30 ± 0.046 and 195.54 ± 0.046 mg gallic acid equivalent/g dry weight of each extract, respectively. Reducing sugars, particularly fructose, ascorbic acid, and protein, may have an effect on the quantity of total polyphenols. Polyphenols contribute to health-promoting and sensorial properties of fruits and vegetables. Further, polyphenols are responsible for varying structure-dependent stability during processing and shelf-life [36].

The protective role of flavonoids has been reported to be against diabetes and its complications, cancer, and cardiovascular diseases. TFC in ethanol extracts of fruit pulp as well as seed were 43.58 ± 0.010 and 35.28 ± 0.180 mg quercetin equivalents/g dry weight of extract.

LC–MS analysis conducted by Khan and coworkers revealed the presence of various phytocomponents in Ajwa date pulp extract belonging to classes such as carbohydrates, phenolics, flavonoids, and terpenoids. Maltose, catechin, myricetin, quercetin, β-sitoserol, digalacturonic acid, chlorogenic acid, and β-carotene were found to be major molecules [37]. Moreover, the LC-linear ion quadrupole mass spectrometric analysis (LC-MS and LC-MS/MS tandem mass spectrometry) analysis revealed the presence of 3,30-di-O-methyl ellagic acid, 7-methoxyquercetin-O-hexose isomers, caffeic acid, ferulic acid, isomers of quercetin-rutinoside, kaempferol methylether, p-hydroxybenzoic acid, phytol, punicalagin, and quercetin-3-O-glucoside (isoquercitrin) in Ajwa [38]. The most dominant phenolic compounds in Ajwa dates, according to Hamad et al. (2015) [39], were p-coumaric acid, gallic acid, and ferulic acid derivatives. Protocatechuic acid, hydroxybenzoic acid, vanillic acid, gallic acid, isovanillic acid, chlorogenic acid, ferulic acid, isoferulic acid, syringic acid, caffeic acid, hydroxycinnamic acid, and chlorogenic acid as the main phenolic compounds [40]. 

### 4.2. Hydrogen Peroxide (H_2_O_2_) Radical Scavenging

**Figure 2:**
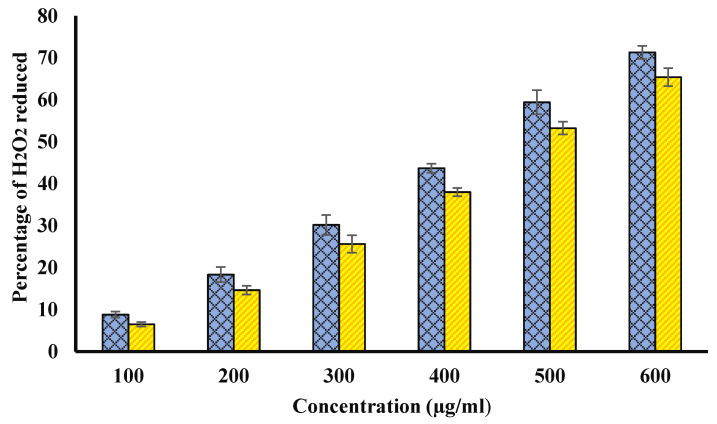
Percentage of H_2_O_2_ scavenging potential of methanol extracts of Ajwa fruit pulp (blue) and seed (yellow). Samples at y-axis show various concentrations of extracts (100, 200, 300, 400, 500, and 600 µg/mL). The results are presented as means ± SEM (*n* = 3, *p* < 0.05).

Reducing capacity and antioxidant activity are directly correlated to each other. Further, there is a significant correlation between antioxidant activity and TPC, hydrogen peroxide scavenging capacity, and DPPH radical scavenging activities. Thus, the assessment of hydrogen peroxide scavenging capacity may be an important indicator to determine the antioxidant activity. The percentage H_2_O_2_ scavenging ability of Ajwa fruit pulp (blue curve) and seed (yellow curve) extracts has been presented in Figure 2. In both cases, H_2_O_2_ scavenging activity was found to be increased in with increase in the concentration of extracts. 

### 4.3. DPPH Radical Scavenging Assay

**Figure 3:**
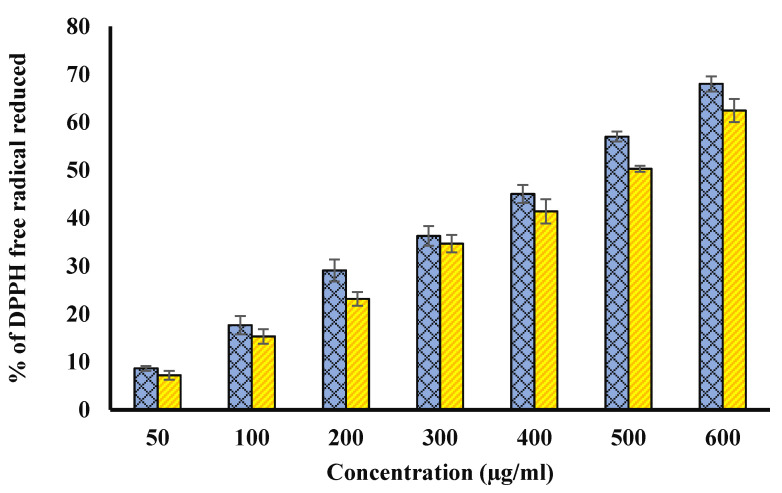
Blue bars show percentage of free radical reduced against fruit pulp concentration (µg/mL). However, yellow columns show the percentage free radical scavenging activity against seed extract concentration (µg/mL) of. The *p*-value significance was found to be less than 0.05 for this figure (*p* < 0.05).

It has been found to be a very high correlation between the concentration of the extracts of natural products and percentage of DPPH radical scavenging activity (Anwar et al., 2020a). In our study, methanol extracts of Ajwa fruit pulp and seed has showed a concentration dependent DPPH scavenging activity (Figure 3) due to their strong antioxidant nature that was comparable to ascorbic acid. IC_50_ is the concentration of an extract or antioxidant that is essential to decrease DPPH concentration by 50%. The low EC_50_ value indicates that the antioxidant is more active. The EC_50_ value of methanol extracts of Ajwa fruit pulp and seed was 1580.360 ± 0.370 and 1272.610 ± 0.270 µg/mL by plot between concentration of methanol extracts and percent of free radical scavenging activity, separately and intercept = 2.710 and 2.320. The equations were for both curve y = 0.18460x + 2.710, and y = 0.1357 + 2.32 (in both cases, R^2^ > 0.96). Figure 3 compares the percentage of free radical scavenging potential of fruit pulp (blue) and seed (yellow) in a bar plot.

### 4.4. Determination of Protein Denaturation inhibition

**Figure 4:**
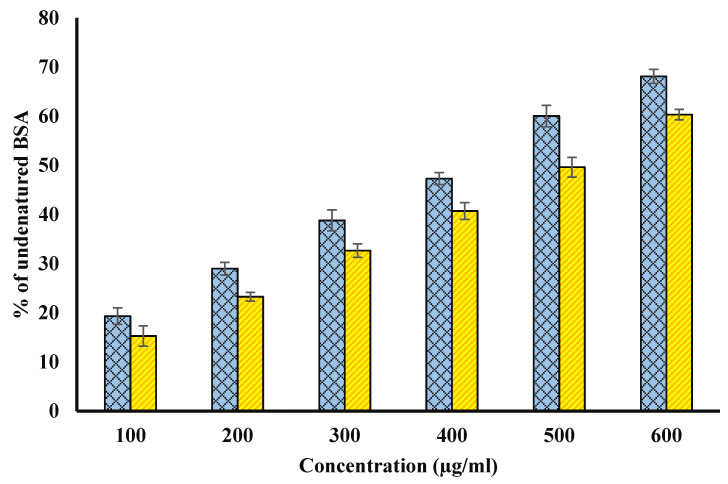
Percentage of undenatured BSA. Blue bars show percentage of undenatured BSA plot for various concentrations (µg/mL) of fruit pulp. However, yellow columns show the percentage of undenatured BSA plot for various concentrations (µg/mL) of seed extract. The *p*-value significance was found to be less than 0.05 for this figure (*p* < 0.05).

During the denaturation process, many weak bonds including hydrogen bonds responsible for native structure of protein become broken. Thus, highly ordered structure of protein become lost. Various factors such as chemicals or stress induce protein denaturation. Besides, protein denaturation contributed to inflammation significantly. As a result, the plausible anti-inflammatory potential of fruit pulp and seed was evaluated by protection from BSA denaturation. The extracts of both pulp and seed were found to be very effective against heat-induced BSA denaturation (Figure 4). Ibuprofen displayed the maximum inhibition, 63.010 ± 0.48% at 200 µg/mL.

### 4.5. Anti-Proteinase Activity

**Figure 5:**
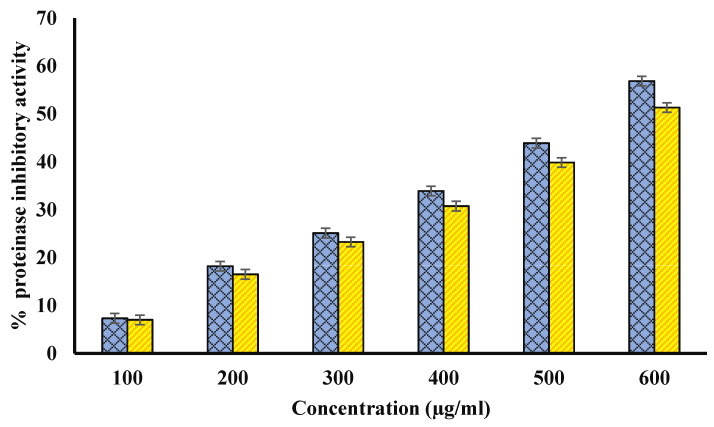
Percentage of proteinase inhibitory activity. Blue bars show percentage of proteinase inhibitory activity plot for various concentrations (µg/mL) of fruit pulp. However, yellow columns show the percentage of proteinase inhibitory activity plot for various concentrations (µg/mL) of seed extract. The *p*-value significance was found to be less than 0.05 for this figure (*p* < 0.05).

Proteinases are known to be involved in arthritic reactions. Both extracts inhibited proteinase activity, and Ajwa fruit pulp and seed methanol extracts (600 μg/mL) showed the highest proteinase inhibition potential of 68 and 60%, respectively, in this study (Figure 5). Diclofenac sodium showed maximum anti-proteinase activity, i.e., 71.59 ± 0.075 at 200 µg/mL.

### 4.6. Inhibition of Egg Albumin Denaturation Inhibition

**Figure 6:**
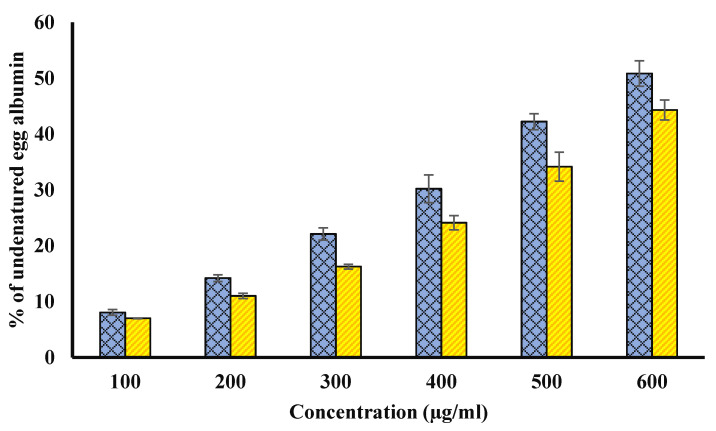
Percentage of undenatured egg albumin. Blue bars show percentage of undenatured egg albumin plot for various concentrations (µg/mL) of fruit pulp. However, yellow columns show the percentage of undenatured egg albumin plot for various concentrations (µg/mL) of seed extract. The *p*-value significance was found to be less than 0.05 for this figure (*p* < 0.05).

The possible anti-arthritic potential of both extracts was evaluated using inhibition of the denaturation of hen egg albumin (Figure 6). The significant ability to protect from heat- induced hen egg albumin denaturation might be due to various non-enzymatic anti-oxidants such as polyphenols. Diclofenac protected from denaturation by 67.010 ± 0.78%, at 200 µg/mL.

### 4.7. Test for Membrane Stabilization Potential

The test for stabilization of the RBCs membrane was performed to further uncover the possible mechanism of anti-inflammatory activity of seed and fruit pulp.

### 4.8. Heat Induced Hemolysis

**Figure 7:**
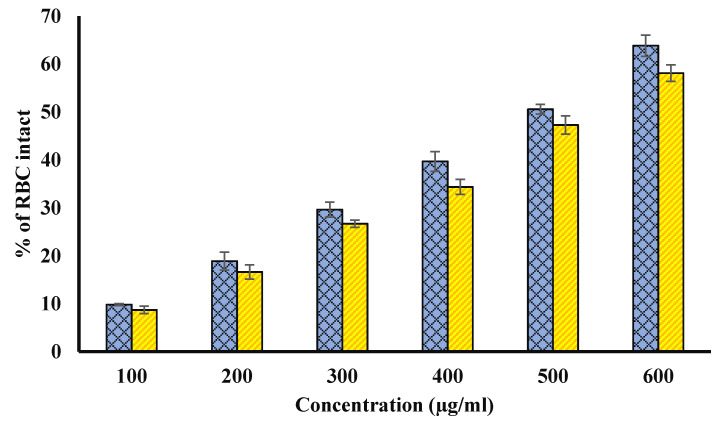
Percentage of RBC intact. Blue columns represent various concentrations (100–600 µg/mL) of Ajwa fruit pulp. Yellow columns represent various concentrations (100–600 µg/mL) of Ajwa seed extract. The results are presented as means ± SEM (*n* = 3, *p* < 0.05).

Both extracts showed a significant (*p* < 0.01) membrane stabilizing potential against hemolysis at all concentrations (Figure 7). Methanol extracts of fruit pulp and seed had highest stabilization of human RBCs membrane (or highest inhibition of hemolysis) by 54.44 ± 0.31 and 50.21 ± 0.63% at 600 µg/mL, respectively, in the current study that was comparable to standard reference drug, i.e., aspirin. Aspirin showed the highest protection from hemolysis by 73.64 ± 0.117% at 200 µg/mL.

### 4.9. Protection from Hypotonicity Induced Hemolysis

**Figure 8:**
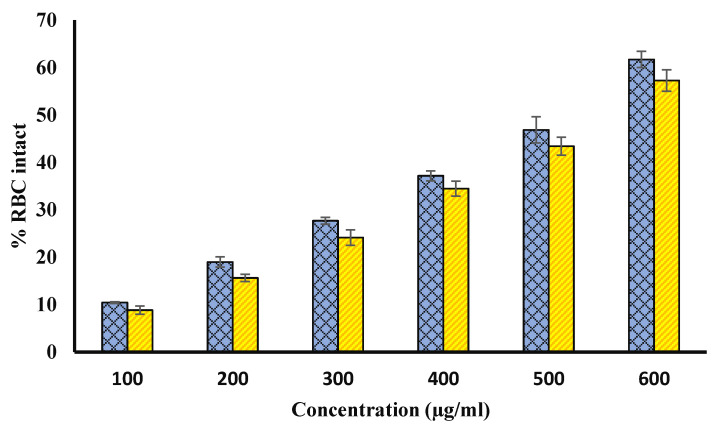
Protection from hyposalinity-induced hemolysis. The figure shows that methanol extract of Ajwa seed and fruit pulp provide protection for hyposalinity-induced hemolysis in a concentration-dependent manner. Blue bars show various concentrations (100, 200, 300, 400, 500, and 600 µg/mL) of Ajwa fruit pulp. Yellow columns represent various concentrations (100, 200, 300, 400, 500, and 600 µg/mL) of seed extract. The results are presented as means ± SEM (*n* = 3, *p* < 0.05).

Extract of Ajwa fruit pulp and seed showed a very good protection for hyposaline- induced hemolysis. Hypotonicity induces hemolysis and contributes to the osmotic loss occurs. In our study, in vitro membrane stabilization was shown by both methanol extracts of Ajwa fruit pulp and seed. Hypotonicity-induced hemolysis was inhibited by both extracts. The percentage inhibition of hemolysis in normal adult erythrocytes by the extract was found to be increased with increase in the concentrations (Figure 8). The standard drug diclofenac sodium with percentage inhibition of 69.34% (at 200 µg/mL) had higher efficacy in inhibiting hypotonic-induced hemolysis of normal erythrocytes.

### 4.10. Effect of Extract on Browning Intensity of Glycated Samples

**Figure 9:**
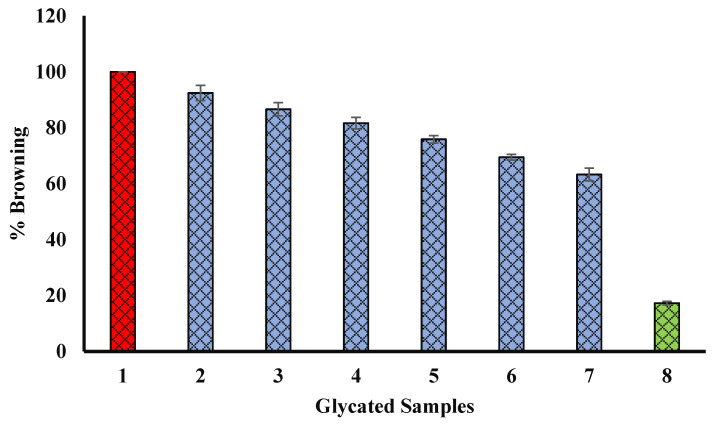
Reduction in % of browning by seed extract. Sample 1 (red column) belongs to BSA kept with glucose for 15 days (100% glycation or browning). Bars 2, 3, 4, 5, 6, and 7 show % of browning of glycated samples with 100, 200, 300, 400, 500, and 600 µg/mL of Ajwa seed extract (blue columns) and % of browning (the degree of glycation) was found to be decreased with an increase in the concentration of seed extract. Bar 8 (green column) shows the % of browning of BSA incubated in the absence of any extract or glucose, and exhibited the least browning (glycation). The results are presented as means ± SEM (*n* = 3, *p* < 0.05).

BSA was glycated by incubating for 15 days at 37 °C with glucose in the absence or presence of seed methanol extract. In our experiment, methanol seed extract was found to have the significant potential to inhibit browning (hence, glycation). The methanol extract of seed exhibited 63.29% browning (at 600 µg/mL) as compared to glycated BSA (BSA incubated with glucose only). The browning intensity of glycated BSA (BSA incubated with glucose only) was supposed to have 100% browning (Figure 9). Our results indicate the presence of extract lead to lesser glycation (or lesser brown products). Hence, browning becomes inhibited in the presence of extracts.

### 4.11. Effect of Seed Extract on Protein Aggregation Index

**Figure 10:**
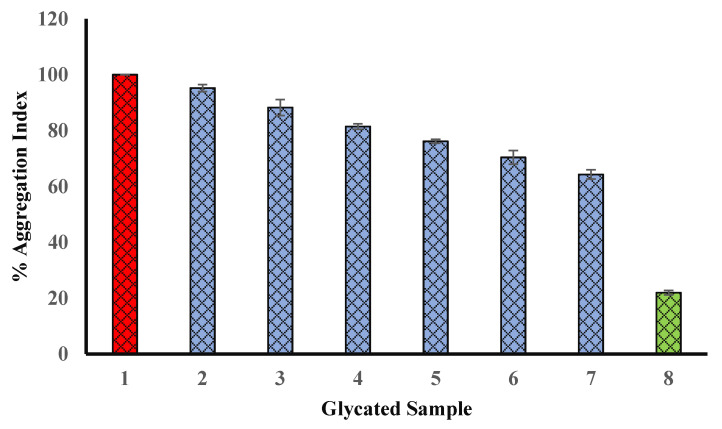
Reduction in percent of aggregation in presence of Ajwa seed methanol extract. Sample 1 (red column) belongs to BSA kept with glucose for 15 days (100% aggregation). Bars 2, 3, 4, 5, 6, and 7 show percent of aggregation of glycated samples with 100, 200, 300, 400, 500, and 600 µg/mL of Ajwa seed extract (blue columns), and the percent of aggregation (the degree of glycation) was found to be decreased with an increase in the concentration of seed extract. Bar 8 (green column) shows the percent aggregation of BSA incubated in the absence of any extract or glucose, and exhibited the least aggregation (glycation). The results are presented as means ± SEM (*n* = 3, *p* < 0.05).

Glycation occurs at N-terminal group or at the side chains of a polypeptide, and it contributes significantly in the modification of protein structure and functions. Further, glycation leads to the formation of aggregates of protein because carbonyl groups linked with protein induce the formation of cluster. There are various reports regarding the involvement of protein aggregates in various diseases and their complications. Many natural products have been reported to protect against aggregate formation in glycated proteins [10,11,12,13,14]. Glycated BSA samples incubated with Ajwa seed extract showed a lesser aggregation index as compared to glycated BSA (Figure 10). 

### 4.12. Congo Red (CR) Assay

**Figure 11:**
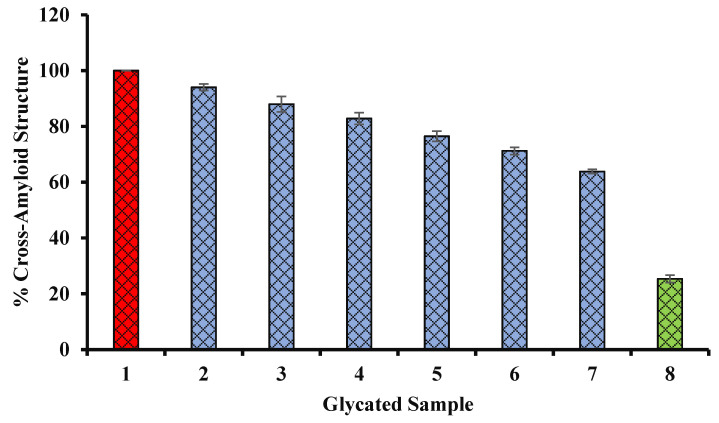
Decrease in cross amyloid structures in the presence of Ajwa seed extract (blue column). Sample 1 (red column) belongs to BSA kept with glucose for 15 days (100% structural modifications). Bars 2, 3, 4, 5, 6, and 7 show percent of structural modifications of glycated samples with 100, 200, 300, 400, 500, and 600 µg/mL of Ajwa seed extract (blue columns), and structural modifications were found to be decreased with an increase in the concentration of seed extract. Bar 8 (green column) shows the structural modifications of BSA incubated in the absence of any extract or glucose, and exhibited the least structural modifications (glycation). The results are presented as means ± SEM (*n* = 3, *p* < 0.05).

The amyloidogenic dye Congo red (CR) effectively interacts with amyloid fibrils and is used to stain amyloid fibrils. Congo red binds to amyloid fibrils of proteins due to several mechanisms including electrostatic interaction with surface residues (positively charged) of the fibril. In aqueous solution, the absorption spectrum of CR shows the highest absorption at 490 nm (blue/green), resulting in a red color solution, at a low concentration and neutral pH. The binding of β-sheet-rich amyloid fibrils to CR is linked with specific orientation of CR dye molecules (i.e., with the long axis of the CR molecules lying parallel to the fibril axis). It causes a red shift (from 490 to 540 nm) in the absorption maximum. In our experiment, the binding of CR was investigated by recording the absorbance of CR-bound glycated samples at 540 nm. The data of the CR binding assay experiment are shown in the Figure 11. Glycated samples with various concentrations of Ajwa seed extract (blue column) indicated a decrease in the fibrillation of BSA. 

### 4.13. Antimicrobial Activity of Seed and Fruit Pulp Extract

Several studies have documented the positive correlation between antioxidant activities of date fruits and antibacterial activities, and they have been shown against common bacterial food pathogens and important disease pathogens. In our experiment, we tested the antimicrobial activity of date fruit pulp extract as well as seed extract of both Gram-negative bacteria and Gram-positive bacteria. The methanol seed extract exhibited the highest inhibitory zone against all tested bacterial strains. However, the fruit pulp did not show a notable inhibitory zone to these bacterial strains and Candida. Seed extract showed notable antimicrobial activity at 100 mg/mL. The inhibition induced by a plant extract against a particular organism was determined by several factors including both external as well as internal factors. The diameter of the zone of inhibition by extract of Ajwa seed against different organisms is shown in Table 3. MIC values are provided in Table 4.

### 4.14. Receptor–Ligand Interaction Study by Molecular Docking

In molecular docking studies, we studied the molecular interaction of necessary antioxidant enzymes, superoxide dismutase (SOD 1 complexed with isoproterenol (PDB id: 5YTU) and catalase catalase3-amino-1,2,4-triazole complex (PDB id: 1DGH)) with the different active constituents of Ajwa date fruit, including 3,3′-Di-O-methylellagic acid, 7-methoxyquercetin-O-hexose isomers or rhamnetin, caffeic acid, ferulic acid, quercetin-rutinoside, 6″′-malonylicariin or kaempferol methyl ether, 4-hydroxybenzoic acid, phytol, punicalagin, and quercetin-3-O-glucoside or isoquercitrin. 

**Figure 12:**
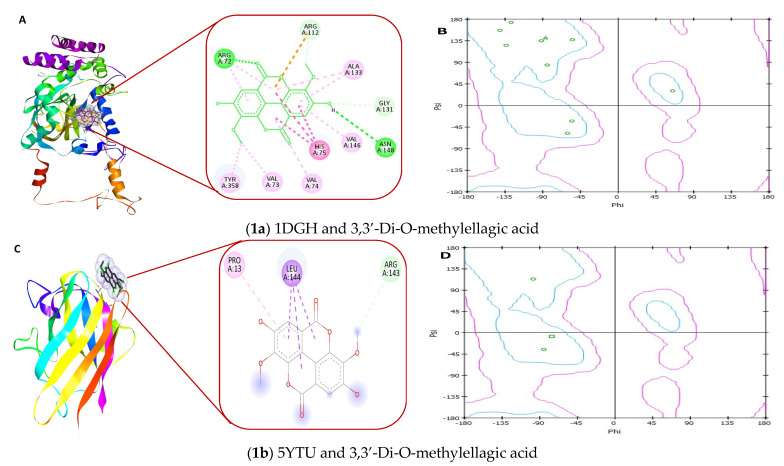

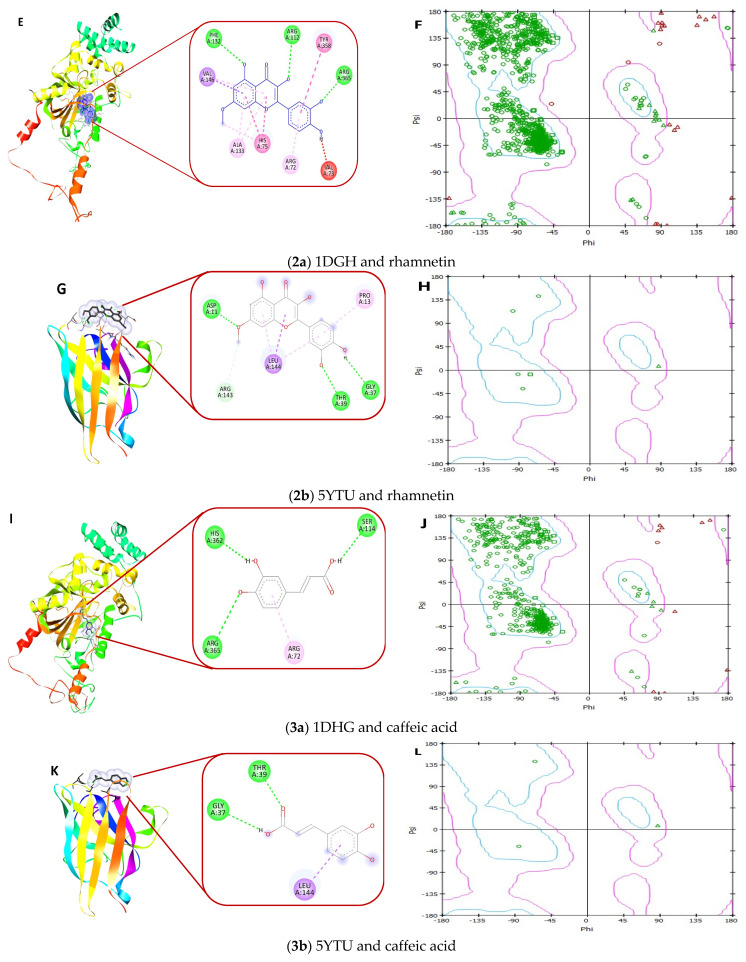

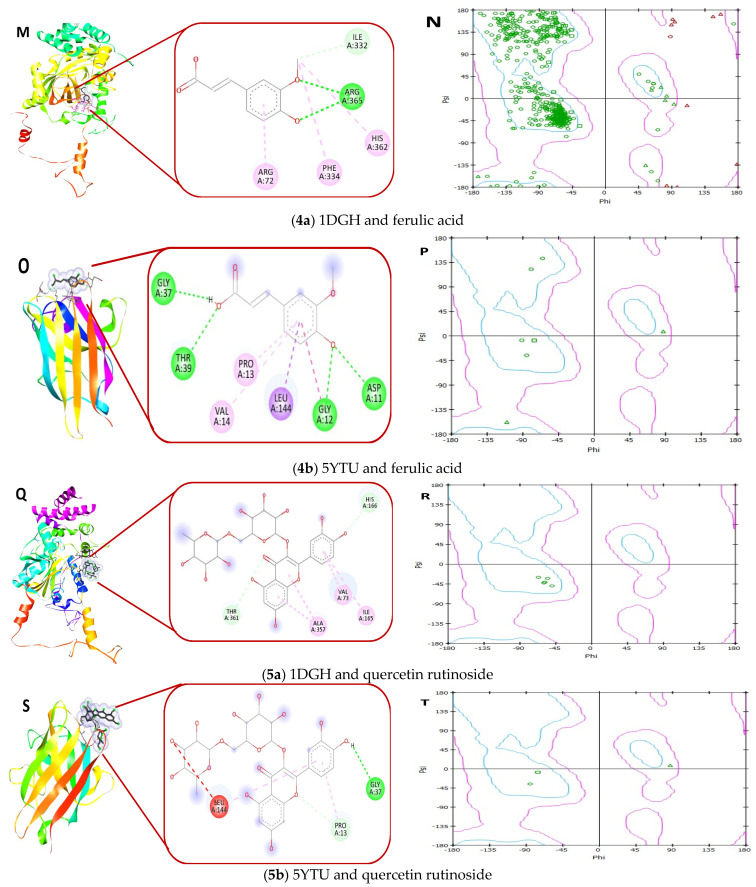

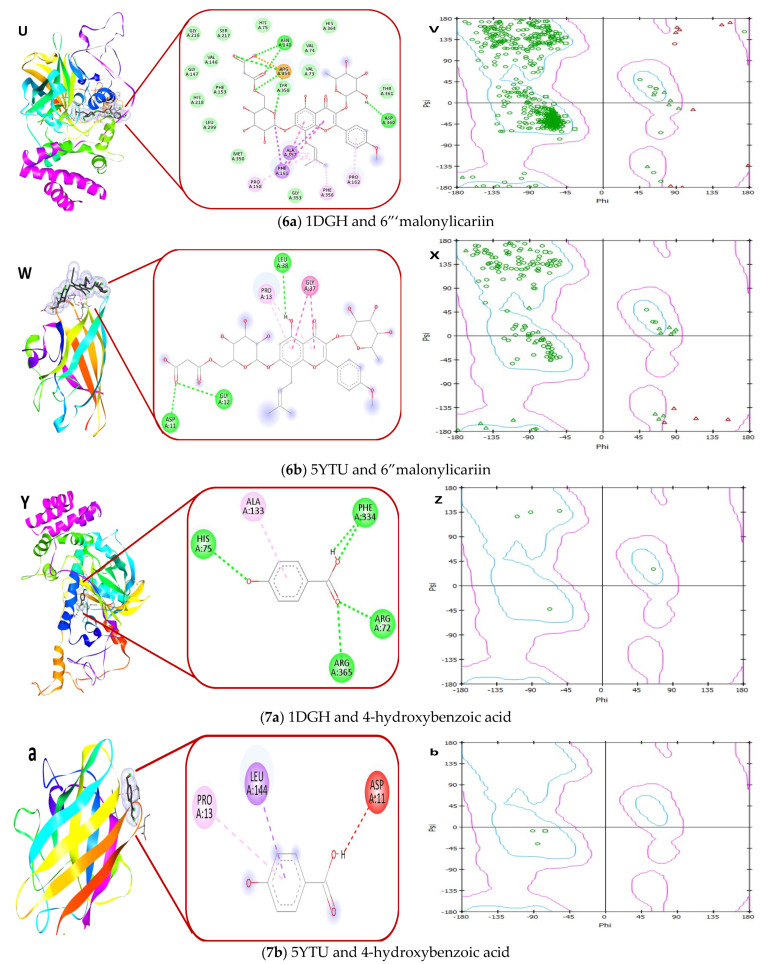

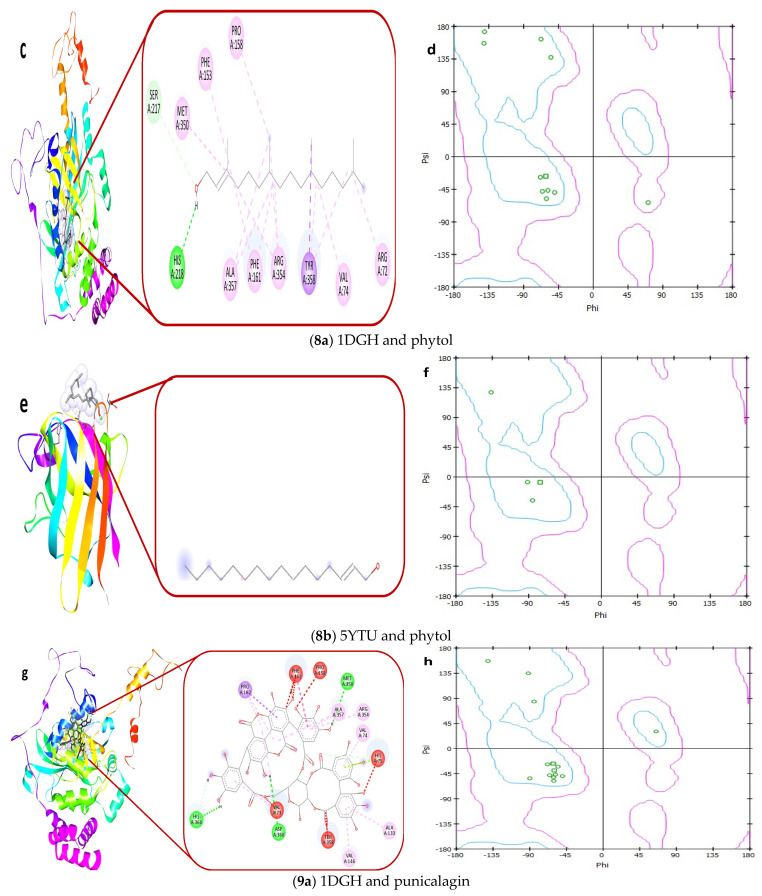

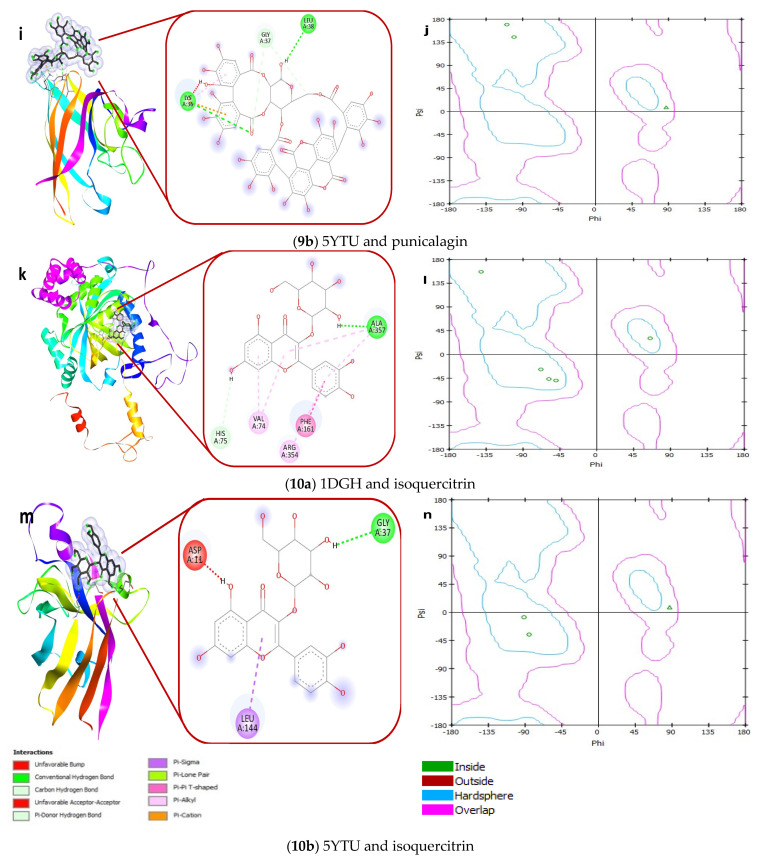
The molecular docking studies of antioxidant enzymes catalase (1DGH) and superoxide dismutase (5YTU) with active constituents of Ajwa date fruit with ligand (**1**) 3,3′-Di-O-methylellagic acid (**A**,**C**); ligand (**2**) rhamnetin (**E**,**G**); ligand (**3**) caffeic acid (**I**,**K**), ligand (**4**) ferulic acid (**M**,**O**), ligand (**5**) quercetin-rutinoside (**Q**,**S**); ligand (**6**) 6′′′-malonylicariin (**U**,**W**), ligand (**7**) 4-hydroxybenzoic acid (**Y**,**a**), ligand (**8**) phytol (**c**,**e**); ligand (**9**) punicalagin (**g**,**i**); ligand (**10**) isoquercitrin (**k**,**m**) and the Ramachandran plot of respective interactions are provided with each ligand-protein interaction (**B**,**D**,**F**,**H**,**J**,**L**,**N**,**P**,**R**,**T**,**V**,**X**,**Z**,**b**,**d**,**f**,**h**,**j**,**l** and **n**).

Based on the results, all the ligands considered for docking analysis of the Ajwa date can bind with both enzymes superoxide dismutase (5YTU) and catalase (1DGH), except ligand punicalagin (Figure 12g). The ligand punicalagin showed binding energy of 19.7 kcal/mol with 1DGH. Details of molecular dockings analysis, such as the name of ligands, PubChem CIDs, binding energy, and interacting amino acids interacting with receptors 1DGH and 5YTU, are shown in Table 5. Figure 12 shows molecular interactions of antioxidant enzymes 1DGH and 5YTU with ligands (active molecules) of the Ajwa date. The primary functional residues in the interactions with 1DGH are amino acids Arg72, Ala357, and Leu144, while with 5YTU, the amino acids are Gly37, Pro13, and Asp11. The A-chain of enzymes is also observed with the interacting amino acids, which is denoted with the “A” with the interacting amino acids. The different types of interactions of the ligand and interacting amino acid of the receptor molecules are shown with different colors; such as the conventional hydrogen bond (dark green), carbon-hydrogen bond (green), Pi-hydrogen bond (light green), Pi-lone pair (faint green), Pi-cation (orange), Pi-Pi (pink), Pi-alkyl (light pink), Pi-Sigma (purple), and unfavorable acceptor–acceptor bump (red) (Figure 12). 

The Ramachandran plot shows the statistical distribution of the combinations of the backbone dihedral angles Phi (ϕ) and Psi (ψ). In practice, the distribution of the Phi/Psi values observed in a protein structure can be used for structure validation. The Ramachandran plot visualizes energetically allowed and forbidden regions for the dihedral angles [41]. The Ramachandran plot analysis of both the enzymes with Ajwa date ligands suggests that most residues are in favored areas (B, D, F, H, J, L, N, P, R, T, V, X, Z, b, d, f, h, j, i, and n). 

## 5. Discussion

Most diseases lead to a decrease in antioxidant defense, thromboembolic consequences, systemic hyper-inflammatory reactions, and the production of reactive oxygen species (ROS), neutrophil invasion, resulting in lysosomal membrane permeabilization. The release of ROS into the cytosol activates of the NLRP3 inflammasome. The NLRP3 inflammasome has been implicated in a number of inflammatory conditions through activation of caspase-1 and release IL-1 [42]. Regarding the substantial morbidity and mortality associated with different diseases including diabetic patients, there are no definite treatments or entirely preventive strategies. As a result, there is an increasing need to uncover the pathobiological processes linked with elevated risk for different diseases and infections to develop a therapeutic strategy.

Various natural products including fruits and their seeds have been used in complementary and alternative medicine (CAM) to protect or alleviate stress, sickness, and to lessen or avoid adverse effects and symptoms, or manage or treat diseases [43]. Fruit wastes are another significant source of high-value bioactive compounds [44]. Date fruit and seed are both considered to be rich sources of various nutrients. Date fruit and seed are both considered as a part of nutrition. Date fruit flesh is solely consumed since it is edible, and the seed half is thrown away as trash. Date seed, on the other hand, has been claimed to be even more healthy, having high nutrients, excellent fatty acids, and a high energy value [45]. A date seed extract has been further claimed to have protective effects against toxicity caused by reactive oxygen species. This inhibitory activity against various reactive oxygen species may be linked with antioxidants present in the extract [19,46].

The receptor, angiotensin-converting enzyme 2 (ACE2), expression is enhanced in patients with diabetes and hypertension who are treated with ACE inhibitors or angiotensin II receptor blockers. In addition, Sartore proposed that glycation of ACE2 has a direct impact on the pathomechanism of COVID-19, and 34 lysine residues are accessible. However, at least one of these residues leads to hydrogen bond’s interaction with the receptor-binding domain (RBD) [47]. Furthermore, the tertiary structure of the protein is altered as a result of the glycation of ACE2 molecule residues. This modification adds to the overexpression or variation in activity of receptor. According to several studies, glycation of CD147 enhances the production of metalloproteinases leading to the relaxing of lung tissue and facilitating the invasion of the virus [48]. Our data show that Ajwa seed methanol extract reduced the browning intensity, aggregation index, and amyloid structures as compared to control samples. Hence, Ajwa seed extract can reduce the complications linked with diabetes, and glycation is occurring in COVID-19 patients. 

A damaged endothelium caused by diabetes may predispose a sufferer to more severe complications and infections, as it has been suggested that the endothelial cells in a similar manner to respiratory cells use the ACE2 receptor, causing inflammation in these cells in patients with COVID-19 infection because inflammatory changes affect the endothelium throughout the body [49]. However, Ajwa seed and fruit pulp extract has exhibited a significant reduction in the heat-induced and hyposaline-induced denaturation of proteins and stabilized the HRBC membrane. Thus, Ajwa can be helpful in stabilizing the membranes and the reduction of the denaturation of proteins associated with inflammatory diseases including SARS-CoV-2 infection. 

Oxidative stress has an impact on repair processes as well as the immunological control system. As a result, oxidative stress is a key contributor to pathogenesis. It proposes that antioxidant supplements should be included in different disease treatment methods. There is a strong relationship between pro-inflammatory components and reactive oxygen species (ROS) in many diseases, including SARS-CoV-2 infection, which is linked with inflammation and oxidative stress [50]. Hydroxyl free radicals are amongst the most prevalent ROS and have been linked to a variety of fatal illnesses, including cancer. The production of hydroxyl free radicals in the blood caused by the breakdown of H_2_O_2_ has already been linked to DNA damage and the promotion of carcinogenesis. Furthermore, hydroxyl radicals have the ability to activate oncogenes such as C-Raf-1 and K-ras [51]. In our study, Ajwa seed and fruit pulp extract have been found to have significant antioxidant potential by DPPH scavenging and H_2_O_2_ reducing activities. Therefore, Ajwa seed and fruit pulp extract can be helpful against most diseases. 

Our data show that Ajwa seed extract has excellent antibacterial activity against both gram-positive and negative bacteria. Antioxidant enzymes make an important defense strategy against the pathology of several diseases. However, glycation may deactivate these enzymes [10,11]. Catalase and superoxide dismutase are important antioxidant enzymes of the human body. The deficient level of these enzymes has been reported to contribute significantly to oxidative stress-induced damage susceptibility of various important tissues and cells like pancreatic β-cells. Ajwa fruit pulp and seed contain several bioactive compounds. The mechanisms of Ajwa accountable for the prevention of diseases still have not been identified but are probably linked to their specific structures. To verify the mode of action of the potential chemical constituents of Ajwa in preventing oxidative stress and denaturation of antioxidant enzymes, and to link the promising molecules of Ajwa to their chemical characteristics, we performed a molecular docking study. In our molecular docking analysis, we took ten bioactive compounds of Ajwa as ligands and investigated the possible interaction of these ligands with two antioxidant enzymes including superoxide dismutase and catalase. Our docking study proved the interaction of these ligands and enzymes. Thus, these ligands might have the capability to protect these enzymes from denaturation caused by glycation. This further confirms our hypothesis that Ajwa seed and fruit extract may protect from oxidative stress in different diseases. Thus, the results of molecular docking studies verify the role of Ajwa in preventing disease progression.

In the most recent viral outbreaks of COVID-19 and SARS-CoV, there are several scientific reports on the positive benefits of traditional Chinese medicine (TCM), and certain polyphenolic substances have been suggested in this regard. Moreover, the antiviral effectiveness of TCM herbal extracts can be ascribed to their active components including baicalein and quercetin that are supposed to decrease COVID-19 by several possible mechanisms [52] such as decreasing NF-*κ*B signaling and 3CLpro activity [53]. Our study confirms the health beneficial effects of Ajwa against the pathophysiology of various diseases that might be involved in the severity and mortality linked with SARS-COV-2 infection in diabetic conditions through in vitro studies. Therefore, the consumption of whole fruit along with seed extract of locally cultivated date palm (Ajwa variety) can be recommended regarding disease prevention. However, it is very important to identify and validate the mechanisms of Ajwa seed ingredients in modulating these diseases.

## 6. Conclusions

The in vitro experiment shows that Ajwa fruit pulp and seed extract possess antioxidant activities, show excellent protective potential against heat-induced albumin and egg albumin denaturation, and stabilize HRBC membrane. Besides, the seed extract inhibits glucose-mediated browning of BSA and cross-amyloid structures formation, AGE formation, and has antibacterial activities. Further, the molecular docking study proved the interaction between human antioxidant enzymes constituents of Ajwa fruit pulp and seed constituents that target the functional residues. Thus, Ajwa components may help in fighting oxidative stress-mediated complications, even in COVID-19 patients. In a nutshell, we can conclude that Ajwa fruit and seed are involved in various health-promoting mechanisms that may be beneficial against the severity of different disease management. 

## Figures and Tables

**Table 1:** Preliminary screening of Ajwa fruit pulp and seed extract.

Preliminary Screening	Ajwa Fruit Pulp	Ajwa Seed
Weight of dry powder of rhizome	100 g	100 g
Yield	18.79%	21.19%
Extract	Methanol	Methanol
Color	Reddish brown	Brown
Odour	Sweet	No specific
Texture	Sticky	Sticky
Flavonoid (alkaline reagent test)	+	+
Phenolic compounds (FeCl_3_ test)	+	+

**Table 2:** Phytochemical screening of Ajwa fruit pulp and seed extract.

Phytochemical Constituents	Fruit Pulp	Seed
Alkaloids	+	+
Saponins	+	+
Tannins	ND	ND
Flavonoids	+	+
Glycosides	+	+
Terpenoids	+	+
Phenolic compounds (FeCl_3_ test)	+	+

**Table 3:** Zone of inhibition against different bacterial strains in the presence of seed extract.

Test Organisms	Seed Extract (The Diameter of the Zone of Inhibition in mm)	
	For 50 mg/mL	For 100 mg/mL
*S. aureus*	13	19
*E. coli*	10	15
*K. pneumoniae*	12	16
*P. aeruginosa*	9	14
*E. faecalis*	11	16

**Table 4:** MIC value against of seed extract different bacterial strains.

Test Organisms	MIC Value of Seed Extract (mg/mL)
*S. aureus*	25
*E. coli*	25
*K. pneumoniae*	25
*P. aeruginosa*	50
*E. faecalis*	25

**Table 5:** Details of molecular docking study; the ligands, binding energy, and interacting amino acids interacting with receptors (1DGH and 5YTU).

Enzymes		Catalase (1DGH)		Superoxide Dismutase (5YTU)	
Ligands	PubChem CID	Binding Energy (kcal/mol)	Interacting Amino Acids	Binding Energy (kcal/mol)	Interacting Amino Acids
3,3′-Di-O-methyl ellagic acid	5488919	−9.9	Arg72, Arg112, Asn148, Gly131, His75, Tyr358, Val73, Val74, Val146, Ala133	−5.1	Arg143, leu144, Pro13
Rhamnetin	5281691	−10.4	Phe132, Arg112, Arg365, Val73, Val146, His75, Tyr358, Ala133, Arg72	−5.8	Asp11, Thr39, Gly37, Arg143, Leu144, Pro13
Caffeic acid	689043	−7.3	His362, Ser114, Arg365, Arg72	−5.0	Thr39, Gly37, Leu144
Ferulic acid	445858	−7.4	Arg365, Ile332, Arg72, Phe334, His362	−4.6	Gly37, Thr39, Gly12, Asp11, Leu144, Pro13, Val14
Quercetin rutinoside	124221768	−4.7	His166, Thr361, Ala357, Val73, Ile165	−6.1	Gly37, Pro13, Leu144
6′′′-Malonylicariin	135398032	−8.5	Asn148, Tyr358, Asp360, Ala357, Phe161, Pro158, Phe356, Pro162	−5.7	Asp11, Gly12, Leu38, Gly37, Pro13
4-Hydroxy benzoic acid	135	−6.5	His75, Phe334, Arg72, Arg365, Ala133	−4.0	Leu144, Pro13, Asp11
Phytol	5280435	−8.2	His218, Ser217, Tyr358, Met350, Phe153, Pro158, Ala357, Phe161, Arg354, Val74, Arg72	−4.1	Asp11, His43, Pro13, Leu144
Punicalagin	44584733	19.7	His364, Asp360, Met350, Pro162, Ala357, Arg354, Val74, Ala133, Val146	−5.6	Leu38, Lys36, Gly37
Isoquercitrin	5280804	−8.6	Ala357, His75, Phe161, Arg354, Val74	−5.4	Gly37, Leu144, Asp11

## Data Availability

All data generated or analyzed during this study are included in this published article.
